# Innate immune signaling and immunothrombosis: New insights and therapeutic opportunities

**DOI:** 10.1002/eji.202149410

**Published:** 2022-05-24

**Authors:** Tristram A. J. Ryan, Luke A. J. O'Neill

**Affiliations:** ^1^ School of Biochemistry and Immunology Trinity Biomedical Sciences Institute Trinity College Dublin Dublin 2 Ireland

**Keywords:** coagulation, immunothrombosis, inflammasomes, STING, tissue factor

## Abstract

Activation of the coagulation cascade is a critical, evolutionarily conserved mechanism that maintains hemostasis by rapidly forming blood clots in response to blood‐borne infections and damaged blood vessels. Coagulation is a key component of innate immunity since it prevents bacterial dissemination and can provoke inflammation. The term immunothrombosis describes the process by which the innate immune response drives aberrant coagulation, which can result in a lethal condition termed disseminated intravascular coagulation, often seen in sepsis. In this review, we describe the recently uncovered molecular mechanisms underlying inflammasome‐ and STING‐driven immunothrombosis induced by bacterial and viral infections, culminating in tissue factor (TF) activation and release. Current anticoagulant therapeutics, while effective, are associated with a life‐threatening bleeding risk, requiring the urgent development of new treatments. Targeting immunothrombosis may provide a safer option. Thus, we highlight preclinical tools which target TF and/or block canonical (NLRP3) or noncanonical (caspase‐11) inflammasome activation as well as STING‐driven TF release and discuss clinically approved drugs which block key immunothrombotic processes and, therefore, may be redeployed as safer anticoagulants.

## Graphical abstract

Detection of pathogens, such as bacteria and viruses, by pattern recognition receptors (PRRs) triggers activation of components of immune cell defense pathways such as the inflammasome, type I interferon, and STING. This culminates in proinflammatory cell death, termed pyroptosis, and release of the coagulation activator, tissue factor (TF). This process of immune cell activation triggering coagulation, termed immunothrombosis, is a hallmark of coagulopathies such as sepsis and disseminated intravascular coagulation.

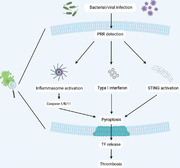

## Introduction

Coagulation is a core component in maintaining physiological hemostasis and the host response to infection. The coagulation cascade is defined by two major pathways—the intrinsic and extrinsic pathways—which culminate in a common pathway which ultimately results in formation of a thrombus and fibrin clot, stopping bleeding. The intrinsic pathway, which primarily contributes to pathological clot formation [1], is initiated via injury to blood vessels by autoactivation of coagulation factor (F)XII upon exposure of plasma to a diverse range of blood‐borne artificial or pathological surfaces, including negatively charged endogenous activating surfaces such as RNA, DNA, polyphosphate, and/or components of atherosclerotic plaques [2]. The extrinsic pathway is initiated by coagulation FIII, also called tissue factor (TF) or CD142, which is expressed at low, basal levels in a complex with FVII on the membrane of circulating immune cells and cells in the blood vessel wall [3, 4, 5]. Blood clotting is controlled by endogenous anticoagulants such as tissue factor pathway inhibitor (TFPI), activated protein C, or antithrombin [6]. However, under pathogenic circumstances, exposure to, and detection of, microbes by innate immune cells amplifies the procoagulant activity of TF up to 100‐fold, resulting in clot formation with the dual role of preventing bleeding but also inhibiting the dissemination of the provoking pathogen [7, 8]. Exposure to bacteria or viruses is detected by pattern recognition receptors (PRRs) on immune cells, such as monocytes, macrophages, endothelial cells (ECs), neutrophils, and platelets, triggering TF production and release. TF is released from macrophages, ECs, and neutrophils via inflammasome‐mediated pyroptosis [9, 10]. This activates the coagulation cascade, restoring, and maintaining hemostasis via rapid development of a thrombus, or blood clot, and subsequent clearance of the pathogen. Thrombin in turn feeds back to drive further inflammation via cleavage of protease‐activated receptors (PARs) and activation of the proinflammatory cytokine IL‐1α [11, 12]. Thus, inflammation and coagulation are innately connected, evolutionarily conserved processes. This interplay has been termed immunothrombosis [13]. Dysregulated immunothrombosis, termed thromboinflammation, characterizes life‐threatening conditions, such as sepsis and disseminated intravascular coagulation (DIC), but also acute respiratory distress syndrome, stroke, myocardial infarction, venous thromboembolism, and coronavirus disease 2019 (COVID‐19) [13, 14, 15, 16, 17, 18].

Coagulopathies, including sepsis and DIC, are conditions of significant microvasculature damage and multiorgan failure, and are the primary cause of death in intensive care units [19]. Appropriately, the World Health Organization has recently recognized sepsis as a global health priority [20], with 48.9 million cases of sepsis and 11 million associated deaths reported in 2017, accounting for just under one‐fifth of all global deaths [21]. In this review, we will describe the critical role of TF in initiating immunothrombosis, and focus on recent developments describing novel mechanisms by which bacterial‐ and viral‐induced immunothrombosis can be triggered via PRRs. In addition, we will discuss new approaches toward targeting these pathways that drive immunothrombosis and thromboinflammation, as a means to treat coagulopathies.

## Tissue factor: The initiator of trauma‐induced coagulation

**Figure 1:**
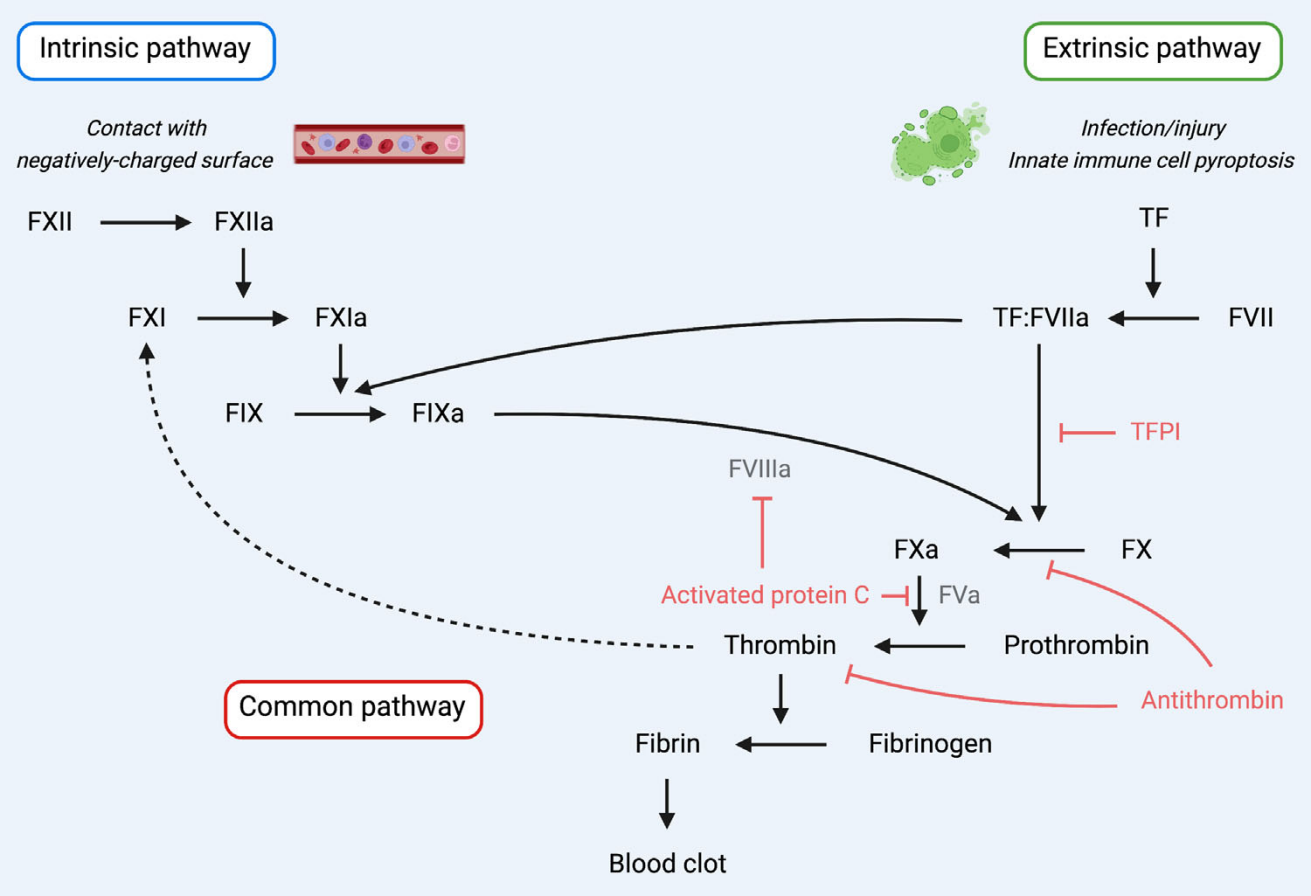
**Two major pathways of coagulation converge during hemostasis to form a blood clot**. The original “waterfall” model of the coagulation cascade comprises the intrinsic and extrinsic pathways which converge into a common pathway to generate thrombin and form a fibrin clot. The intrinsic pathway primarily contributes to pathological clot formation and is activated when FXII encounters blood‐borne, negatively charged surfaces such as RNA, DNA, and components of atherosclerotic plaques. The extrinsic pathway is activated when subvascular TF is exposed to plasma, or released into the bloodstream via innate immune cell pyroptosis, where TF forms a cell‐surface complex with FVIIa. The intrinsic and extrinsic pathways combine to activate FX, which drives thrombin generation and ultimately blood clot formation. Endogenous inhibitors of the coagulation cascade include TFPI, activated protein C, and antithrombin.

TF is a 47‐kDa membrane glycoprotein and receptor and the key trigger of infection‐ and injury‐induced coagulation [5, 22–24]. TF is critical for survival, as deletion in mice leads to universal embryonic death [25, 26, 27], and defects in TF gene expression are associated with differing clinical outcomes in patients with severe sepsis [28]. TF is expressed by adventitial tissues, such as ECs, and blood‐borne circulating immune cells such as monocytes, macrophages, and neutrophils [5]. During hemostasis, blood vessel injury triggers exposure and release of extravascular TF into the bloodstream, where it forms a complex with FVII and contributes to blood clotting via low‐level activation of the extrinsic pathway of the coagulation cascade, before rapid inhibition by TFPI (Fig. 1) [4]. However, detection of pathogen‐associated molecular patterns (PAMPs), such as LPS by PRRs such as TLR4, triggers immunothrombosis via rapid induction of TF at the mRNA level. This occurs via PAMP‐induced activation of the transcription factor NF‐κB both in vitro and in vivo [29], in monocytes and macrophages [29, 30, 31], neutrophils [32, 33], ECs [34, 35], and epithelial cells [36], the primary sources of TF [37].

TF is modified in a process termed decryption, which occurs in‐part via changes in the lipid composition in the outer leaflet of the cell membrane [8, 10, 38], increasing the procoagulant activity of TF [7, 8]. Decrypted TF is then released from immune cells through inflammasome‐induced pyroptotic pores, via activation of the NOD‐, LRR‐, and pyrin domain‐containing protein 3 (NLRP3, via caspase‐1) or noncanonical (via caspase‐11) inflammasomes [9, 10]. The molecular mechanisms underlying this process have recently been studied in detail in monocytes and macrophages, as they are the main source of circulating TF [23, 37]. For example, deletion of monocytes and macrophages using clodronate or gadolinium chloride significantly attenuates thrombin generation and septic shock‐induced mortality in mice in vivo [9, 10].

**Figure 2:**
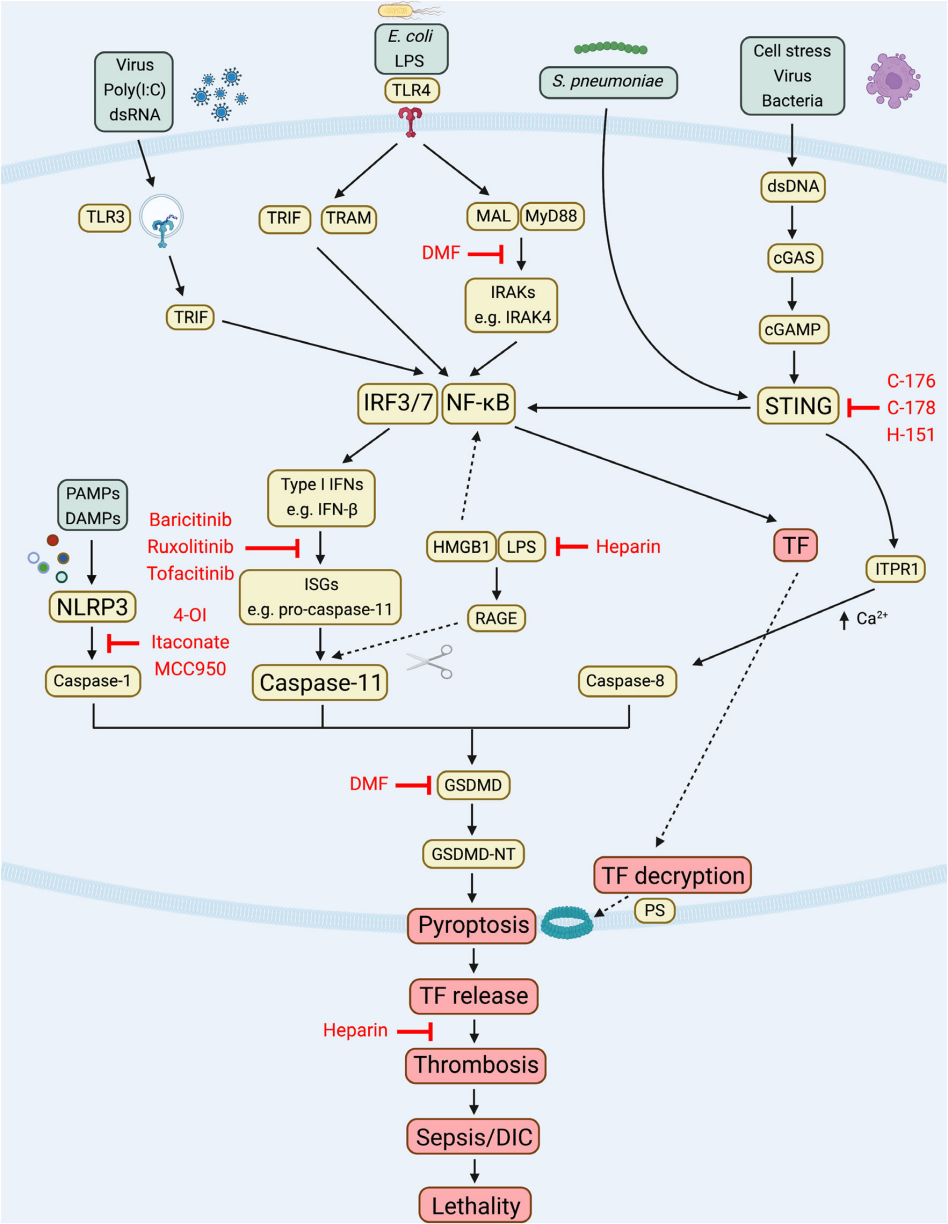
**Inflammasome‐ and STING‐mediated TF release drives thrombosis**. Detection of a diverse range of microbes (such as viruses and Gram‐negative and Gram‐positive bacteria) by PRRs triggers innate immune signalling cascades which converge to activate IRF3/7 and NF‐κB. IRF3/7 stimulates expression of type I IFNs. This leads to IFN‐β release, which acts via the JAK‐STAT signalling complex to drive transcription of hundreds of ISGs including caspase‐11. Activation of STING can also drive this process. Caspase‐11 is then cleaved and activated upon recognition of cytosolic LPS (which occurs via HMGB1 and RAGE), triggering cleavage and activation of GSDMD, resulting in pyroptosis. GSDMD cleavage can also be triggered by caspase‐1 or caspase‐8 activation. Simultaneously, TF is induced by NF‐κB, before TF is post‐translationally activated, in a process termed decryption. Procoagulant TF is then released through the pyroptotic pores to drive thrombosis, which can result in thromboinflammation, sepsis, and disseminated intravascular coagulation. These signalling cascades have been shown to be blocked by a number of immunomodulatory compounds including DMF, heparin, STING inhibitors (C‐176, C‐178, H‐151), JAK inhibitors (Baricitinib, Ruxolitinib, Tofacitinib), and NLRP3 inflammasome inhibitors (4‐OI, Itaconate, MCC950). Thus, innate immune signalling can trigger TF‐mediated thrombosis via activation of the inflammasome and STING.

Following its release via pyroptotic pores, decrypted TF is expressed in the circulation on outer membrane vesicles [39, 40, 41, 42] and forms a high‐affinity cell‐surface complex with FVII/VIIa to proteolytically activate factors IX to IXa and X to Xa, resulting in thrombin generation [5, 43]. Thrombin then activates PARs which are critical for the interplay between inflammation and coagulation, boosting proinflammatory cytokine secretion but also activating platelets [44, 45]. Thrombin also cleaves fibrinogen to fibrin which generates a clot by forming a mesh at the site of infection, in conjunction with activated platelets and neutrophils which expel their DNA, histones, and granule‐derived enzymes to form networks of extracellular fibres called neutrophil extracellular traps (NETs), in a process termed NETosis [46, 47, 48, 49, 50]. NETs then propagate thrombosis by capturing TF and TF‐positive extracellular vesicles from the circulation, further driving coagulation [51, 52]. Thus, detection of PAMPs by PRRs triggers induction and decryption of TF, increasing its procoagulant activity, which is the key initiating step in coagulopathy associated with immunothrombosis and thromboinflammation (Fig. 2).

## Inflammasomes and TF

Caspase‐11 (in mice; caspase‐4/5 in humans) is a member of the evolutionarily conserved family of caspases that mediate cell death [53]. It is induced and activated in response to Gram‐negative bacteria, but not Gram‐positive bacteria [54]. The response of caspase‐11 to Gram‐negative bacteria forms what has been termed as a noncanonical inflammasome. LPS induces transcriptional upregulation of caspase‐11 in a range of immune and nonimmune cells including macrophages, neutrophils, and ECs [14, 53, 55–59]. Activation, and subsequent cleavage, of caspase‐11 occurs upon detection of cytosolic LPS [56, 60, 61], triggering proteolytic cleavage of gasdermin D (GSDMD), a member of the family of gasdermin proteins that cause cell death [57, 62]. The pore‐forming, *N*‐terminal fragment of GSDMD is released, inserting into the cell membrane to form large oligomeric pores [63]. This leads to a proinflammatory, lytic form of cell death, termed pyroptosis, as first identified by Kayagaki et al. in a seminal paper in 2011 [55]. Pyroptosis, therefore, provides a critical host defense mechanism by killing infected cells and preventing dissemination of a pathogen.

Caspase‐1 forms a canonical inflammasome and processes the proinflammatory cytokines IL‐1β and IL‐18. NLRP3 is a key activator of caspase‐1 and is stimulated upon exposure to a diverse range of pathogens [64] via potassium efflux [65]. Caspase‐1 is then recruited to the complex and autoproteolytically activated where it cleaves GSDMD, forming sublytic pores in the cell membrane [64, 66, 67].

Canonical and noncanonical inflammasome activation has recently been shown to be critical for the release of TF from immune cells. It had previously been reported that caspase‐11 is highly expressed in primary human macrophages in patients with severe sepsis [68], hinting at its importance in immunothrombosis. In 2019, Wu et al. showed that activation of both the canonical (with EprJ type III secretion system rod proteins from *Escherichia coli* (*E. coli*)) and noncanonical (with LPS) inflammasomes in macrophages triggers TF release via pyroptosis, leading to severe thrombosis and lethality [9]. Deletion of caspase‐11 and TLR4 (but not caspase‐1) in mice did not affect EprJ‐induced caspase‐1 cleavage and TF release, whereas deletion of both caspase‐11 and ‐1 blocked TF release, highlighting the requirement for caspase‐1 in pyroptosis and TF release [9]. Injection of mice with clodronate‐containing liposomes, which depletes macrophages, significantly reduced EprJ‐induced plasma levels of thrombin‐antithrombin and fibrinogen (which are markers of TF‐mediated thrombosis [15]), as well as lethality [9]. Another 2019 study supported these findings, showing that activation of caspase‐11 and GSDMD is essential for LPS‐induced thrombosis [10]. Notably, GSDMD increased the procoagulant activity of TF via externalization of phosphatidylserine (PS) [10], a cell membrane phospholipid that is mostly expressed on the inner cell membrane during homeostasis [5, 8, 39]. This GSDMD‐mediated increase in TF activity occurs via influx of calcium into the cell [10]. This is consistent with reports from the 1980s and 1990s that PS and calcium are key regulators of TF decryption, and thus, enhance TF‐initiated coagulation [8, 39, 40]. Furthermore, in a mouse model of blood flow restriction‐induced venous thrombosis, deletion of caspase‐1 and GSDMD, but not caspase‐11, protected mice against venous thrombosis [69]. Deletion of macrophages, using gadolinium chloride, also protected against venous thrombosis [69]. These studies directly implicated inflammasome‐mediated macrophage cell death as a trigger of immunothrombosis in response to NLRP3 activation, cytosolic LPS, and in ischemia.

## cGAS‐STING and immunothrombosis

Recently, activation of the DNA sensor cyclic GMP–AMP synthase (cGAS)‐STING has been implicated as a driver of sepsis in models of human and mouse coagulopathies. In 2014, mutations in transmembrane protein 173 (*TMEM173*) (the gene which encodes STING) were found to increase production of IFN‐β in PBMCs and fibroblasts from pediatric patients presenting with recurrent fevers, ulcerative skin lesions, vasculitis, and interstitial lung disease, in addition to systemic inflammation, cutaneous vasculopathy, and pulmonary inflammation [70, 71]. ECs, which express STING, were also found to increase IFN‐β production when stimulated with the second messenger cyclic guanosine monophosphate–adenosine monophosphate [70]. Furthermore, TF expression was upregulated in vascular ECs from patients with a mutation in *TMEM173* [70]. These reports, describing a severe autoinflammatory syndrome termed STING‐associated vasculopathy with onset in infancy, were the first to link STING with a coagulopathy.

STING has been shown to sustain the host procoagulant response at later timepoints by regulating calcium release from macrophages and monocytes to drive GSDMD cleavage, facilitating the release of TF [72]. Notably, however, Zhang et al. found that this occurs in a type I IFN‐independent manner [72]. This occurs in monocytes and macrophages via binding of STING with inositol 1,4,5‐trisphosphate receptor type 1 (ITPR1), the primary calcium release channel from the ER. The authors found that a STING‐ITPR1 complex forms after infection with the Gram‐negative bacterium *E. coli*, or the Gram‐positive bacterium *Streptococcus pneumoniae* (*S. pneumoniae*), which activates caspase‐8. STING‐ITPR1 binding boosts release of calcium from the ER into the cytosol, triggering cleavage of GSDMD via activation of caspase‐1/11 (after *E. coli* infection) or caspase‐8 (after *S. pneumoniae* infection). This facilitates pyroptosis and subsequent release of TF, resulting in sepsis and DIC [72]. The authors concluded that this process was type I IFN‐independent as deletion of IFNAR, the type I IFN receptor, did not significantly alter mouse blood coagulation markers, such as platelets, fibrinogen, d‐dimer, and TF, when assayed 48 h after caecal ligation and puncture (CLP)‐induced sepsis [72]. Furthermore, stimulation of human and mouse monocytes and macrophages with IFN‐α and IFN‐β did not induce TF release, whereas stimulation with *E. coli* and *S. pneumoniae* both induced TF release [72]. This highlights the specificity of pathways that drive coagulation within certain contexts. Two key signals are required for inflammasome‐mediated coagulation: the first signal is infection‐ or injury‐associated induction of TF at the mRNA and protein levels; the second signal is activation and cleavage of inflammatory caspases to trigger pyroptosis and release of procoagulant TF. After infection with *E. coli* or *S. pneumoniae*, TF is induced rapidly at the mRNA level via NF‐κB, in addition to activation of caspase‐1/11/8‐mediated pyroptosis, representing the two key signals of inflammasome‐mediated coagulation. However, when cells are stimulated with IFN‐β, there is no known direct induction of TF mRNA via NF‐κB.

Contrastingly, Yang and Cheng et al. showed a critical role for type I IFN signalling as a driver of coagulation in mouse models of LPS‐ and CLP‐induced septic shock. In this study, the authors assessed coagulation markers between 6 and 16 h after infection, and found that the deletion of IFNAR significantly reduced LPS‐induced plasma levels of thrombin‐antithrombin and d‐dimer, in addition to increasing survival of mice [73]. This was verified using TIR‐domain‐containing adaptor‐inducing interferon‐β (TRIF) KO mice, which were also protected against LPS‐induced septic shock [73]. The different timepoints used in these two studies may explain their differing conclusions, but may also point toward type I IFNs driving a procoagulant phenotype at the onset of infection or injury, while STING may directly trigger coagulation at later timepoints after the type I IFN response has peaked.

## HMGB1 and immunothrombosis

The danger‐associated molecular pattern, high‐mobility group box protein 1 (HMGB1), has been linked with coagulation as it is increased in the serum of LPS‐infected mice and septic patients [74]. In addition, HMGB1 expression on circulating platelets is increased in trauma patients [75]. Recent studies have found that HMGB1 derived from platelets, hepatocytes, and myeloid cells mediates LPS‐induced thrombosis in mice in a TLR4‐ and MyD88‐dependent manner [75, 76, 77]. HMGB1 contributes to Gram‐negative sepsis by binding to LPS [78], and it has been shown that hepatocyte‐released HMGB1 transports extracellular LPS into the cytosol of macrophages and ECs [79]. This occurs via endocytosis of HMGB1‐LPS, mediated by the receptor for advanced glycation endproducts (RAGE), and subsequent HMGB1‐induced rupture of the endolysosomal membrane, releasing LPS into the cytosol. Cytosolic LPS is then detected by caspase‐11, triggering noncanonical inflammasome‐induced pyroptosis, releasing TF to drive coagulation [79].

HMGB1 has also been shown to stimulate expression of TF in vitro at the mRNA and protein levels in vascular ECs and macrophages via activation of the transcription factors NF‐κB and Egr‐1 [80]. However, Yang and Cheng et al. did not see an effect on LPS‐induced TF protein levels in vivo after deletion of IFNAR, TRIF, or hepatocyte HMGB1 [73]. Using KO mice, they surmised that type I IFN and extracellular HMGB1 drive procoagulant TF activation and coagulation post‐transcriptionally via caspase‐11‐ and GSDMD‐triggered pyroptosis and subsequent exposure of PS (which decrypts TF to trigger coagulation) [73]. In addition, a recent study assessing the role ninjurin1 (Ninj1) in lytic cell death found that deletion of Ninj1 in macrophages impaired pyroptosis and release of HMGB1, highlighting the importance of cell membrane rupture in driving inflammation and coagulation via release of HMGB1, and likely, TF [81].

Therefore, it is possible that extracellular LPS stimulates caspase‐11‐TF‐induced coagulation initially by activating NF‐κB (and inducing TF at the mRNA level), while simultaneously, extracellular LPS also drives type I IFN‐mediated induction of IFN‐stimulated genes (ISGs) such as caspase‐11. LPS is then delivered to the cytosol via HMGB1, cleaving and activating caspase‐1 (inducing sublytic pores in the cell membrane) and caspase‐11, which triggers lytic pyroptosis and TF release. HMGB1 might then feedback to induce further TF expression, amplifying the available procoagulant TF. Furthermore, as the type I IFN response subsides, STING might then sense bacterial or host‐derived DNA, driving TF release by regulating changes in calcium, activating GSDMD‐induced pyroptosis. Further in vivo studies are required to unravel the differing roles of these key players in immunothrombosis.

## Virally‐induced immunothrombosis

Induction and decryption of TF has been shown to occur in vitro and in vivo in response to a range of viruses and the viral ds RNA mimic polyinosinic:polycytidylic acid (poly[I:C]) [82, 83, 84]. TF procoagulant activity is increased in ECs infected with Herpes simplex virus (HSV) [85]. HSV infection in ECs also stimulates increased thrombin generation and platelet activity [86]. Ebola virus infection is also associated with severe hemorrhagic complications, manifesting as DIC which is driven by TF activity [87]. Geisbert et al. showed that TF is increased at the mRNA and protein levels in PBMCs from macaque monkeys infected with Ebola virus, with TF‐positive microvesicles also increased in plasma from infected macaques [87]. A follow‐up study from Geisbert et al. found that inhibition of TF:FVIIa, using recombinant nematode anticoagulant protein c2, following exposure to Ebola virus, significantly reduced coagulation, the cytokine storm, and mortality in rhesus monkeys [88]. Infection of ECs with Dengue virus also induces NF‐κB‐mediated TF expression [89].

Furthermore, HIV is associated with an increased risk of thrombosis. TF expression on the surface of monocytes is increased in humans infected with HIV [90]. Expression of TF in HIV patients correlates with plasma levels of d‐dimer and soluble CD14, the LPS receptor that is released by monocytes after LPS stimulation in vivo [90]. TF expression in human ECs is also increased after infection with Zika virus, boosting thrombin generation [91], which likely contributes to the coagulopathy associated with Zika virus infection [92]. However, further studies are required to decipher the relative roles of immunothrombotic regulators within innate immune cells, such as cGAS‐STING and/or type I IFN, and perhaps as yet unidentified mechanisms, in driving TF induction and release upon viral infection.

Severe acute respiratory syndrome coronavirus 2 (SARS‐CoV‐2) also drives a profound coagulopathy associated with COVID‐19, which is triggered by the key players of immunothrombosis [18]. SARS‐CoV‐2‐infected ECs release von Willebrand factor [93], promoting inflammation and coagulation by attracting platelets and neutrophils to the site of infection. Neutrophil activation and subsequent release of NETs is increased by SARS‐CoV‐2 infection [94, 95]. NETs then capture TF and TF‐positive microvesicles, triggering activation of the coagulation cascade [51, 52, 96, 97]. TF and TF‐positive microvesicles are also increased in ECs and epithelial cells from patients with severe COVID‐19 [98, 99], propagating the coagulopathy associated with COVID‐19 infection, with TF‐positive microvesicles a clinical marker of severity in patients with COVID‐19 [100, 101]. This may be due to SARS‐CoV‐2‐induced activation of the canonical NLRP3 and noncanonical caspase‐11 inflammasomes [102, 103], resulting in TF release via pyroptosis. Thus, COVID‐19 has been termed a syndrome of dysregulated immunothrombosis [104].

## Targeting immunothrombosis to prevent coagulopathies

Current clinically approved anticoagulant therapies, while highly effective, are associated with increased risk of bleeding because blood clotting, platelet aggregation, and fibrin cross‐linking are essential during normal hemostasis [105, 106, 107, 108, 109]. This life‐threatening bleeding risk is significantly increased with treatment of sepsis and DIC [110]. Anticoagulant therapies exert their function by decreasing activity of clotting factors in the common pathway of the coagulation cascade. The widely used anticoagulant heparin exerts its anticoagulant function by activating antithrombin, which in turn inactivates thrombin, FXa, and FIXa [111]. Intriguingly, it has recently been shown that heparin, or a chemically modified form of heparin without anticoagulant function, also blocks HMGB1‐mediated cytosolic delivery of LPS, thus, inhibiting caspase‐11‐driven pyroptosis to prevent aberrant immunothrombosis and subsequent sepsis‐induced lethality in mice [112]. This hints at a potential solution to the bleeding risk associated with existing anticoagulant drugs and an exciting prospect for the development of new anticoagulant therapies: could targeting both PRR‐mediated induction of TF and/or inflammasome activation within immune cells, rather than clotting factors themselves, prevent coagulopathy while also eliminating the associated bleeding risk?

Might inhibition of the transcriptional processes that lead to inflammasome activation and pyroptosis be particularly attractive targets in this context? PAMP‐induced type I IFN and JAK‐STAT signalling is required for expression of ISGs such as caspase‐11. Baricitinib, ruxolitinib, and tofacitinib are clinically approved JAK inhibitors for the treatment of rheumatoid arthritis and myeloproliferative neoplasms [113], and thus, potentially could be redeployed as inhibitors of immunothrombosis. Recently identified STING inhibitors, such as the nitrofurans (C‐176 and C‐178) [114, 115], indole ureas (H‐151) [114], and the acrylamides (BPK‐21 and BPK‐25) [116], which covalently modify STING, might also be useful. Notably, a recent study showed that ex vivo treatment with H‐151 blocked induction of TF mRNA in primary human ECs infected with SARS‐CoV‐2 [99]. In addition, H‐151 reduced lung SARS‐CoV‐2‐induced TF mRNA levels in a mouse model of COVID‐19 [99].

Directly targeting inflammasome activation is another strategy that has been shown to reduce immunothrombosis in several models. MCC950 is a highly selective inhibitor of NLRP3 [117, 118] and attenuates platelet activation and multiorgan injuries in a rat model of CLP‐induced sepsis [119]. Similarly, the endogenous, Krebs cycle‐derived metabolite itaconate, and its potently anti‐inflammatory cell‐permeable derivative, 4‐octyl itaconate (4‐OI), also block NLRP3 activation [120], with 4‐OI attenuating lung injury in a murine model of LPS‐induced coagulopathy [121]. This warrants further testing of these preclinical inhibitors of the canonical (NLRP3) and noncanonical (caspase‐11) inflammasomes as potential treatments for inflammasome‐driven immunothrombosis. Inhibition of GSDMD activation and pyroptosis occurs following treatment with dimethyl fumarate (DMF) [122, 123]. DMF is a clinically approved drug for the treatment of multiple sclerosis and psoriasis, and it exerts its immunomodulatory effects in‐part by blocking induction of type I IFN [124] and inhibiting NLRP3 activation in a murine experimental colitis model via activation of the regulatory transcription factor Nrf2 [125]. Activation of Nrf2 is protective in a model of LPS‐ and NF‐κB‐induced sepsis [126], which would further support the testing of DMF as an anti‐immunothrombotic agent, as TF‐driven thrombosis occurs via activation of NF‐κB. As such, DMF is currently being investigated as a potential broad spectrum anti‐inflammatory therapy for COVID‐19 in the ongoing RECOVERY trial [127].

## Clinically approved anti‐inflammatory therapies as potential anticoagulants?

Recent clinical trials have also studied the effects of anti‐inflammatory therapies on thrombosis (*discussed in detail in* Refs. [18, 109]). The anti‐inflammatory drug, colchicine, utilized for the treatment of gout and pericarditis, significantly lowered the risk of ischemic events in the COLCOT trial when administered to patients after myocardial infarction [128]. Colchicine blocks immunothrombosis by inhibiting NET formation and can also attenuate NLRP3 activation [129, 130]. A follow‐up trial, LoDoCo2, using low‐dose colchicine, found that IL‐18 and myeloperoxidase (an enzyme released during neutrophil activation) were markedly decreased when administered to patients with chronic coronary disease [131, 132], highlighting the importance of drug dosing in anticoagulation treatment. However, a limitation of colchicine is that it is renally excreted, and thus, can be toxic in patients with chronic kidney disease [133], restricting its use as a treatment for cardiovascular diseases.

## Concluding remarks

The past decade has seen a flurry of research in the area of immunothrombosis. As targeting mediators of the coagulation cascade downstream of inflammasome activation and pyroptosis has not yielded any new, safer anticoagulant drugs [134], developing therapeutics that inhibit immunothrombosis during activation of the innate immune response to infection, for example, by blocking TF expression and/or inflammasome or STING activation and subsequent pyroptosis, presents an exciting prospect. As this occurs prior to the activation of the coagulation cascade and generation of thrombin, the anti‐inflammatory agents described above may in turn provide a safer method of anticoagulation by preventing any risk of unwanted bleeding, which has been termed the Holy Grail of identifying new treatments for immunothrombosis [135]. In the interim, redeployment of clinically approved anti‐inflammatory drugs for the safer treatment of aberrant coagulation might well be a highly effective way to prevent the coagulopathies associated with immunothrombosis.

## Conflict of interest

The authors declare that there is no conflict of interest associated with this manuscript.

## Author's contribution

T.A.J.R. wrote the original draft. L.A.J.O'N. critically reviewed and edited the manuscript.

**Abbreviations**
4‐OI: 4‐octyl itaconate
cGAMP: cyclic guanosine monophosphate–adenosine monophosphate
cGAS: cyclic GMP–AMP synthase
CKD: chronic kidney disease
CLP: caecal ligation and puncture
COVID‐19: coronavirus disease 2019
DAMP: danger‐associated molecular pattern
DIC: disseminated intravascular coagulation
DMF: dimethyl fumarate
EC: endothelial cell
*E. coli*
: *Escherichia coli*
GSDMD: gasdermin D
HMGB1: high‐mobility group box protein 1
HSV: Herpes simplex virus
IFNAR: IFN‐α/β receptor
ISG: IFN‐stimulated gene
ITPR1: inositol 1,4,5‐trisphosphate receptor type 1
NET: neutrophil extracellular trap
NINJ1: ninjurin1
NLRP3: NOD‐, LRR‐ and pyrin domain‐containing protein 3
PAR: protease activated receptor
poly(I:C): polyinosinic:polycytidylic acid
PS: phosphatidylserine
RAGE: receptor for advanced glycation endproducts
SARS‐CoV‐2: severe acute respiratory syndrome coronavirus 2
STING: stimulator of interferon genes
*S. pneumoniae*
: *Streptococcus pneumoniae*
TF: tissue factor
TFPI: tissue factor pathway inhibitor
TMEM173: transmembrane protein 173
TRIF: TIR‐domain‐containing adaptor‐inducing interferon‐β

## Data Availability

Data sharing not applicable to this article as no datasets were generated or analyzed during the current study.
